# Particle simulation of the strong magnetic field effect on dust particle charging process

**DOI:** 10.1038/s41598-023-28310-y

**Published:** 2023-01-20

**Authors:** Hadi Davari, Bizhan Farokhi, Mohammad Ali Asgarian

**Affiliations:** 1 Department of Physics, Faculty of Science, Arak University, P.O. Box 38156-8-8349, Arak, Iran; 2 Faculty of Physics, University of Isfahan, Isfahan, 81746-73441 Iran

**Keywords:** Particle physics, Plasma physics

## Abstract

A particle-in-cell simulation is modeled and run on a dusty plasma to determine the effect of the magnetic field on the process of dust-particle charging through electron–ion plasma. The electric field is solved through the Poisson equation, and the electron-neutral elastic scattering, excitation, and ionization processes are modeled through Monte Carlo collision method. The effects observed from the initial density of the plasma, the initial temperature of the electrons, and the changing magnetic field are included in this simulation model. In the dust particle charging process, saturation time and saturation charge are compared. An increase in the magnetic field does not reduce time to reach the saturation state. Determining the magnetic field boundaries which depend on the physical properties of the plasma, can be contributive in some areas of dusty(complex) plasma. The applications of the results obtained here for fusion plasma conditions and space and laboratory plasmas are discussed. The results here can be applied in future simulation models with a focus on the dust particle movement and their effect on plasma, leading to the modeling of different astrophysical plasmas thorough laboratory experiments.

## Introduction

Dusty particles are naturally present in many cosmic plasmas and in some laboratory apparatus. Their presence is essential in the production of dusty plasma crystals or in plasmas in assessing the complex plasma behavior. Dusty plasmas consist of neutral atoms, ions, electrons, and charged microparticles. Generally, microparticles are produced from ice, silica, carbon, metals, or dielectric materials, with their radius of typically a few hundreds nm to a few μm. The large mass of dusty particles (compared with that of ions or electrons where m_dust_ > 10^12^ m_i,e_) is contributive in their dynamical effects in dusty plasmas on time scales in a milliseconds order^1^. How the particles are charged and their effect on electrical potential on plasma properties are of major concern among the researchers. There exists a difference among the dust particle, electron, and ion behavior in plasma in presence of magnetic field and their behavior in its absence, which necessitating research in this area with respect the magnetic field strength^2–7^.

The study of dusty plasma behavior has a relatively long history in different aspects of theory, experiments, and simulations. As to the theoretical aspect, researchers in^7^ run a systematic analysis of low-frequency waves like hydro-magnetic and acoustic waves in a magnetized dusty plasma. in the same context, a nonlinear propagation of dust-acoustic waves is assessed in^6^. The effect of external magnetic field on charging currents of electrons and ions to a spherical dust grain and dust charge fluctuation damping in a dusty plasma is assessed in^5^, where it is stressed that the external magnetic field could reduce the charging currents in a magnetized dusty plasma. The dependency of dust charge on the external magnetic field strength is assessed in^4^.

As to the experimental aspect, there are many studies. The contribution of magnetic field in dusty plasma is discussed in^1^ where briefly reviewed a series of experimental studies to demonstrate the benefits of magnetic fields in controlling the background plasma and modifying the confinement and dynamics of the charged micron dust grains. They also designed a Magnetized Dusty Plasma eXperiment (MDPX) apparatus that is being manufactured at Auburn University. The researchers in^8^ have assessed the effect of external magnetic field on the Langmuir probe measurement and dust charging in plasma. Their experiment is runing a dusty plasma apparatus where plasma is generated through the hot cathode filament discharge technique. Where it is assumed that the reduction factor on quasi-neutrality condition is prevalent, they observed that the influence of magnetic field on dust charge is almost negligible in *low magnetic field* cases.

In this context, applying computer simulation methods is advantageous due to the numerous and inexpensive features, compared to the experimental studies. The simulation methods require high computing power and high storage space for computing location, momentum, and energy of 10^25^ particles and storing their information. Thus, alternative numerical computation and simulations are applied instead of direct computation^9^. The first particle models of electrostatic plasma were originated from pioneering works of Buneman^10^, Birdsall, and Bridges^11^. These models are one dimensional and do not apply grid for field computation. Burger^12^ and Hockney^13^ developed 1 and 2D grid models respectively, and their algorithms are applied in the nearest grid point charge assignment and field interpolation to save computer run time. Higher-order interpolation schemes are first applied by Birdsall and Fuss^14^ in Berkley group to reduce the simulation noise^13^. Today, the model presented in DiP3D is used to study the charging of dust particles in an electromagnetic field^15,16^. The DiP3D code is a three dimensional particle-in-cell (PIC) code. In the PIC method the plasma particles (i.e., electrons and ions) interact with each other via computational grid that is used to calculate the force field.

In this article, a series of the simulation is run to observe the effect of externally applied magnetic field on dust charging through particle-in-cell (PIC) code named *XOOPIC*. This code is devised to be capable of simulating dust particles with different mass and charges. The charge accumulated on dust grains at different magnetic field strengths is computed by the capacitance model and modified quasi-neutrality condition in its theoretical sense. A comparison made between theoretical models and the simulation results is presented in the current paper. The effects of different plasma parameters on dust charging are reported in the presence/absence of magnetic field.

The manuscript is organized as follows: The dust particle charging process is presented in Section "Dust particle charging process". The numerical method is proposed in Section "Numerical method", the result and discussion expressed in Section "Results and discussion" and the article is concluded in Section "Conclusion".

## Dust particle charging process

The electric charge of dust particles is highly contributive in assessing plasma in laboratory experiments, in ionosphere layer, and in its interplanetary state. It is assumed that the dust particles are initially charge free, while electrons and ions eventually collide with the dust surface and have a high chance of sticking to them, thus they are being charged. Some factors like photoemission, secondary electron emission, thermionic emission, and electromagnetic fields can contribute to electrically charged dust particle count^17,18^. Orbital motion limited (OML) is considered a common method of tracking the motion direction of electrons and ions, while can influence different forces within the plasma, determine the collisional cross-sections, and compute the electric charge of the dust at equilibrium^19,20^.

Here, it is assumed that dust particle is of a conductive spherical shape, so the electric potential of the dust surface, φ_s_, which depends on the electric charge to the capacitance ratio of the conductive sphere, φ_s_ = Q/C, where, ${\text{C}} = 4\pi \varepsilon_{0} {\text{r}}_{{\text{d}}}$ is the spherical dust capacity^21^. Most electrons, due to their low mass and high temperature, are exposed more to dust particles in relation to ions, producing a negative charge on the dust particle (φ_s_ < 0). The net charge can be positive on the dust, and φ_s_ > 0 by considering other factors like the emission of electrons from the surface of the dust due to light emission. Solving the motion equations for the electrons and ions reveal the intensity of ion and electron flow towards the dust, provided φ_s_ < 0 hold true^22^:

1 $$I_{i} = I_{0i} \left( {1 - \frac{{z_{i} e\varphi_{s} }}{{k_{B} T_{i} }}} \right),$$

2 $$I_{e} = I_{0e} \exp \left( { \frac{{e\varphi_{s} }}{{k_{B} T_{e} }}} \right),$$

otherwise, φ_s_ > 0 for positive dust:

3 $$I_{i} = I_{0i} exp\left( { \frac{{ - z_{i} e\varphi_{s} }}{{k_{B} T_{i} }}} \right),$$

4 $$I_{e} = I_{0e} \left( {1 + \frac{{e\varphi_{s} }}{{k_{B} T_{e} }}} \right),$$

where z_i_ is the ionization degree, T_i_ is the ion temperature, and T_e_ is the electron temperature. The symbols, k_B_ and I_0α_ are the Boltzmann constant and the intensity of the initial current of electrons and ions, respectively:

5 $$I_{0\alpha } = 4\pi r_{d}^{2} n_{\alpha } q_{\alpha } \left( {\frac{{kT_{\alpha } }}{{2\pi m_{\alpha } }}} \right)^{1/2} ,\quad \alpha = e, i,$$

where n_e_ and n_i_ are the electron and ion count per unit volume, respectively, while m_α_ is either e or i of mass, and q_α_ is either e or i of charge. The radius of the dust particles, r_d_, is usually just a few μm, and the charge of the dust particles leads to a balance between electron and ion count.

6 $$\frac{dQ}{{dt}} = I_{e} + I_{i} .$$

By inserting Eq. (5) in Eq. (6), The negative and positive potentials are yield, respectively:

7 $$\frac{dQ}{{dt}} = 4\pi er_{d}^{2} \sqrt {\frac{{k_{B} }}{{2\pi m_{e} }}} \left\{ { - n_{e} \sqrt {T_{e} } exp\left( { \frac{eQ}{{k_{B} CT_{e} }}} \right) + n_{i} z_{i} \sqrt {T_{i} } \left( {1 - \frac{{z_{i} eQ}}{{k_{B} CT_{i} }}} \right)} \right\},$$

8 $$\frac{dQ}{{dt}} = 4\pi er_{d}^{2} \sqrt {\frac{{k_{B} }}{{2\pi m_{e} }}} \left\{ { - n_{e} \sqrt {T_{e} } \left( {1 + \frac{{e\varphi_{s} }}{{k_{B} T_{e} }}} \right) + n_{i} z_{i} \sqrt {T_{i} } exp\left( { \frac{{ - z_{i} eQ}}{{k_{B} CT_{i} }}} \right)} \right\}.$$

Both the Eqs. (7) and (8) are the time evolution of the electric charge of dust particles^23^.

To assess the collisions between dust particles and electrons or ions, The Monte-Carlo method is applied. The electrons and ions have cross-sections of σ_e_ and σ_i_ and energies E_e_ and E_i_, respectively computed through Eqs. (9) and (10) and the immobile dust particles with charge Q_d_ and radius r_d_ are modeled according to OML theory^19^:

9 $$\sigma_{e} = \pi r_{d}^{2} \left( {1 + \frac{{Q_{d} }}{{4\pi \varepsilon_{0} r_{d} E_{e} }}} \right),$$

10 $$\sigma_{i} = \pi r_{d}^{2} \left( {1 - \frac{{Q_{d} }}{{4\pi \varepsilon_{0} r_{d} E_{i} }}} \right)$$

where E_e_ and E_i_ are the electron and ion energy in eV. The cross-sections are subject to the momentum and energy conservation of electrons and ions interacting with dust particles, therefore, the cross-sections are valid for electrons and ions as to they being absorbed or rejected by the dust particles^24^.

The electron–ion collision cross-sections applied in this model are resemble that of^25^. The Coulomb cross-section, σ, for electron and ion scattering by immobile dust particles are extracted from^26^:

11 $$\sigma = \frac{{\pi \left( {e_{\alpha }^{2} e_{\beta }^{2} } \right) ln\Lambda }}{{\left( {\mu v^{2} /2} \right)^{2} }} = \frac{{\pi \left( {e_{\alpha }^{2} e_{\beta }^{2} } \right) ln\Lambda }}{{16\pi^{2} \varepsilon_{0}^{2} \left( {\mu v^{2} /2} \right)^{2} }} = \frac{{Q_{d}^{2} ln\Lambda }}{{16\pi \varepsilon_{0}^{2} E_{\alpha }^{2} }},\quad \alpha ,\beta = e, i,$$

where α and β are the interacting particles, μ is their reduced mass, close to the electron or ion mass because of the existence of large dust-particle mass, lnΛ is the Coulomb logarithm ~ 10, e_α_ and e_β_ are the particle charges, Q_d_ is the dust particle charge, and E_α_ is the electron or ion energy in eV.

## Numerical method

**Figure 1:**
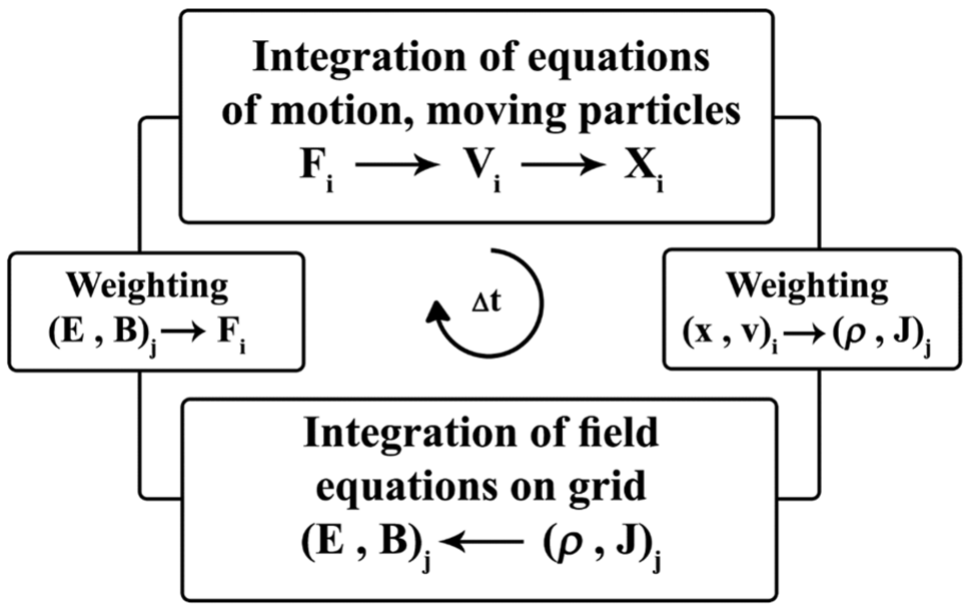
A flow chart of the processes involved in one timestep of the particle-in-cell method. This algorithm is discussed in^27^.

Determining the location, velocity, momentum, and energy of each particle in the plasma is subject to repeating one or more cycles and solving the equations of motion for each particle, according to Fig. 1.

As observed, by repeating this cycle, particles path in time is determined through particle-in-cell (PIC) computational cycle, designed by^27^. The code applied here is based on the development of the OOPIC 2D code^28^, modified to simulate dust particle which if included in plasma is considered as dusty plasma. In the process the particle collision algorithm is considered as a type of Monte-Carlo simulation with random paths based on the cross-section according to Eqs. (9–11). Whether an electron or ion collides, it is decided in this method by comparing a random number with the collision probability at every step. This probability is determined by the collision cross-section. The magnetic field doesn’t change energies of electrons and ions directly. So in this paper, magnetic field effects on the collection cross-sections of electron and ion by dust particles is not assessed. This assessment requires a modification in Eqs. (9 and 10) which will be the subject of the new work by the same authors.

Here, the Ar plasma is applied at P = 1 mTorr pressure. The simulation environment consists of 2D grid cells (Cartesian geometry, 32 × 32 cells) with periodic boundary conditions, a length and width of L_x_ = L_y_ = 2.66 mm, an initial particle density of n_0i_ = n_0e_ = 10^16^ particles/m^3^, ions and dust particles are at room temperature, T_i,d_ = 0.026 eV (300 °K), and electron temperatures T_e_ are on the order of 1–10 eV. The radius, mass and density of carbon dust particles assumed to be r_d_ = 2 μm, m_d_ = 2 × 10^−14^ kg and n_d_ = 1.42 × 10^10^ particles/m^3^ respectively. So we have N_d_ = 100,474 dust particles in which by using super particle definition method one dust super particle is a representation of almost 98 dust particles. They are randomly placed in grid cells with at least one dust particle in every cell. Each simulation’s time step is about Δt = 1 ps, which is smaller < τ_e_ of electrons with a plasma frequency of ω_pe_ = 5.64 × 10^9^ Hz. The magnetic field changes perpendicular to the particle plate from B_z,ext_ = 0 to 50 T. These computations are repeated for different electron densities and temperatures. After the electrons and ions collide directly with the dust particles, they stick to the dust particles, but because they represent only a small proportion of the total particle count, almost < 5%, quasi-neutrality is conserved in this simulation.

## Results and discussion

**Figure 2:**
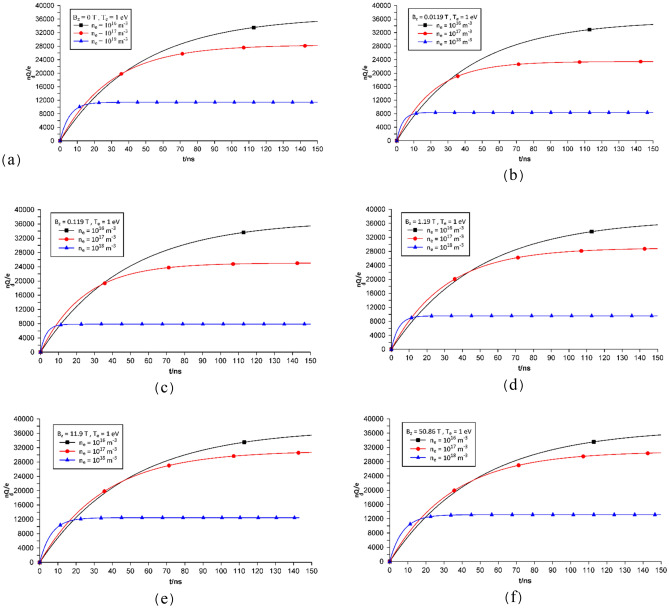
Time evolution of the dust particle charges with the same initial plasma density and temperature, and exposed to different magnetic field (**a**) B_0z_ = 0 T, (**b**) B_0z_ = 0.0119 T, (**c**) B_0z_ = 0.119 T, (**d**) B_0z_ = 1.19 T, (**e**) B_0z_ = 11.9 T, and (**f**) B_0z_ = 50.86 T.

The time evolution of the electric charge of dust particles is shown in Fig. 2, where as observed the external magnetic field is zero, and the electrons have an initial density of 10^16^ m^−3^, 10^17^ m^−3^, and 10^18^ m^−3^. An increase in density of electrons increases the particle count per unit volume, with the probability of collision occurrence between electrons and dust particles, which makes the dust particles to reach the saturation state in a rapid manner, thus, the equilibrium state. This phenomenon reduces the charge volume of the dust particles because they have a less chance in absorbing or losing electrical charge from the plasma. As to the time factor, the time to reach the saturation state for a density of 10^18^ m^−3^ is 10 ns, while for lower densities, of 10^16^ m^−3^, it takes more than 150 ns. At saturation state, it is possible to observe the difference in the dust particles charge volume. Based on the results of the simulation, the time to reach this electric charge threshold decreases with an increase in electron density. At external magnetic field exposure, many important longitudinal scales including the electron cyclotron radius, r_ce_, ion cyclotron radius, r_ci_, radius of dust particles, r_d_, and the collisional cross-section of the electrons or ions with the dust particles, σ_ed_ and σ_id_, can change the accumulation of electric charge on a dust particle.

**Figure 3:**
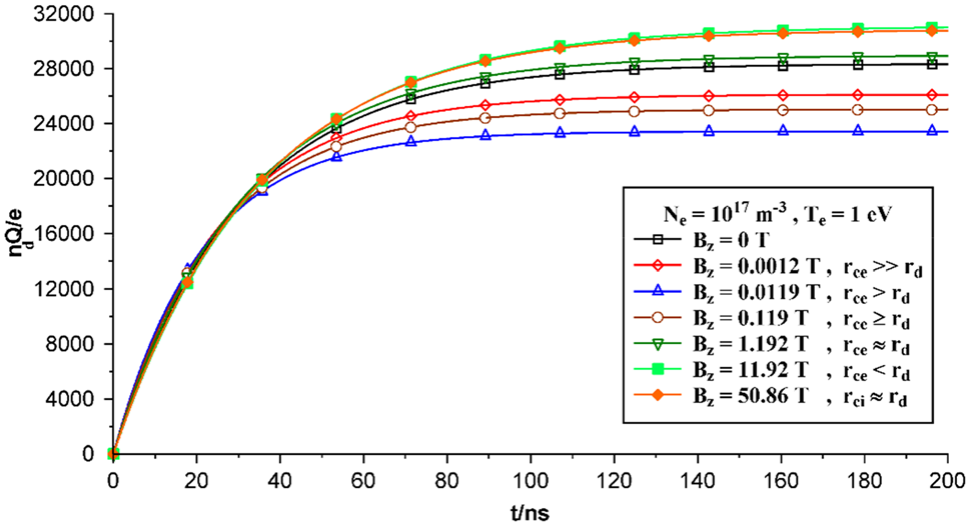
Time evolution of the dust particle charges with the plasma density n_0e_ = 10^17^ m^−3^, magnetic field B_z_ = 0, 0.0012, 0.0119, 0.119, 1.192, 11.92, 50.86 T, and initial electron temperature T_0e_ = 1 eV.

The process of dust-particle charging is shown as a function of time in Fig. 3, where the initial electron density is assumed to be constant and the results are presented based on applying different magnetic fields.

In a weak magnetic field, the electron cyclotron-radius (r_ce_) is smaller than the dust size (r_d_) and the change in dust charge is relatively small. Here, electrons approach the dust grain surface along the external magnetic field direction in a rapid manner, and the fastest in charging a grain may be of Boltzmann distribution, consequently, the OML electron current remains constant. The ions are absorbed by the dust, and their effective cross-section, A_i_, is larger than their geometrical cross-section, πa^2^. Although for weak magnetic fields the dust charge remains the same.

When the magnetic field becomes stronger than a critical value, that is at the electron cyclotron-radius (r_ce_) equal to the collection radius of the electrons on the dust grains (~ λ_De_), only fast magnetized electrons are involved in the charging process, while the rest are rejected backwards along the magnetic field direction. Because the charging cross-section for electrons in presence of magnetic field is smaller than that of magnetic field absence, the magnitude of the electron current volume decreases. If the ion cyclotron-radius (r_ci_) is still smaller than the ion-dust absorption size (~ λ_Di_), the ions are absorbed to the dust grain of approximately the same rate, and their effective cross-section, A_i_, will be greater than the geometrical cross-section, πa^2^. The ion current on the grain will then remain the same as that of a non-magnetized plasma. There exists a direct relation between electron current and dust charge.

In stronger magnetic fields [i.e., B_0_ ≥ (c/ea) (m_i_T_i_)^½^] in a plasma, the ion cyclotron-radius (r_ci_) becomes smaller or comparable with the ion-dust attraction size (~ λ_Di_), where, both the electron and ion currents are modified due to the strong magnetization of the plasma particles.

The time to reach the saturated state for a higher density of electrons and ions decreases because more collision between electrons (ions) and dust particles occur. As collision rate increases, saturation time and the time to take plasma particles to equilibrium state decreases. So reaching a saturation state would be faster than the lower density situation.

**Figure 4:**
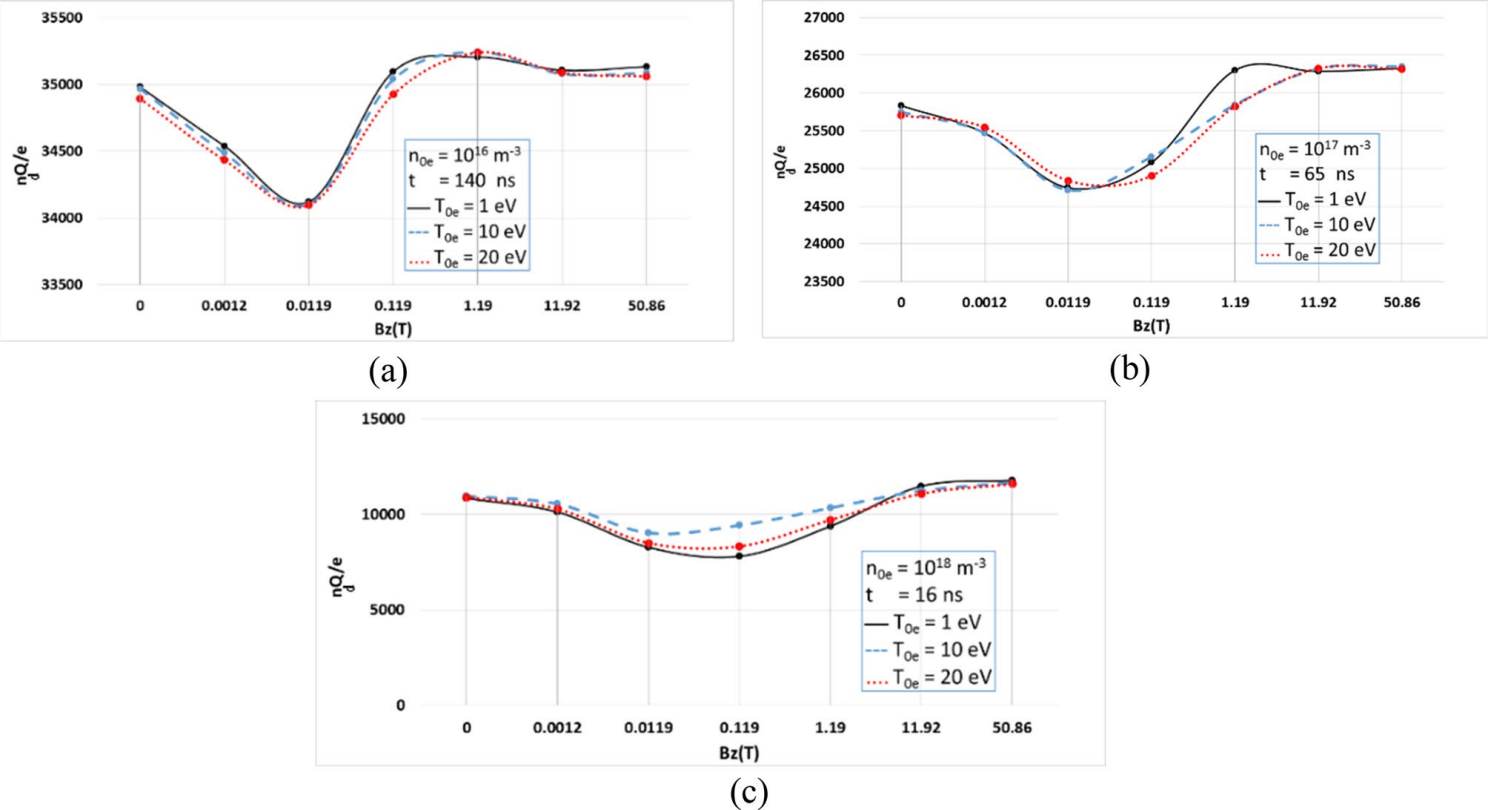
Dust particle charges at saturation time, where exposed to different magnetic fields; B_z_ = 0, 0.0012, 0.0119, 0.119, 1.192, 11.92, 50.86 T, different initial electron temperatures; T_0e_ = 1, 10, 20 eV, and different initial plasma density; (**a**) n_0e_ = 10^16^ m^−3^, (**b**) n_0e_ = 10^17^ m^−3^, (**c**) n_0e_ = 10^18^ m^−3^.

The amount of electric charge of the dust particles at a given time when they approach electric-charge saturation are curved in Fig. 4, where as observed three initial electron temperatures of: 1 eV, 10 eV, and 20 eV with three densities of: 10^16^ m^−3^, 10^17^ m^−3^, and 10^18^ m^−3^; and are applied in B_z_ (T) = 0, 0.0012, 0.0119, 1.192, 11.92, 50.86 T magnitudes of the static magnetic field. At a given time, it is possible to observe the difference in dust particle charge, which is initially high (B_z_ = 0.0 T), while decreases when the magnetic field increases. This allows the electrons to become magnetized, and only fast electrons can reach the dust particles (from B_z_ = 0.0012 T to B_z_ = 0.119 T). After increasing the magnitude of the magnetic field, dust particle charge increases again, indicating the magnetization of the ions at that given time and involve all the electrons in the charging process (from B_z_ = 0.119 T to B_z_ = 50.86 T).

In general, only the application of strong external magnetic field can lead to an increase in the electric charge saturation of the dust particles. These results are consistent with theoretical and experimental findings^2,4,16,23^.

## Conclusion

The effects of a strong magnetic field on the dust charging process are discussed. A newly developed PIC simulation code is applied to simulate the dusty plasma in describing of dust-particle charging process and to predict the saturation state time and charge. It is revealed that, depending on the initial density of the plasma, the time to reach the saturation state varies from 15 to 150 ns. Moreover, since the time to reach the saturation state was several nanoseconds, each time step in the dusty plasma simulations has to be smaller than it, which unlike this study, is not observed in many simulation codes. The time to reach the saturation state is inversely proportional to the initial density of the plasma and the radius of the dust particles. An increase in the initial temperature of the plasma electrons reduces the time necessary to reach the saturation state, where, the dust particles have a negative charge because the electrons highly contribute to the charging process. It is found that an increase in the magnitude of the magnetic field does not necessarily reduce the time to reach the saturation state or more saturation charge while this can be provided by only sufficient strong magnetic field. Finding the boundaries of this magnetic field, which evidently depends on the physical properties of the plasma, can be applicable in many physics and laboratory contexts, like tokamak wall fusion plasma conditions. To decrease complexity, the hypothetical shape for the dust particles is assumed to be spherical, but any shape and size can be applied in simulations. The 3D simulations provide the considerable means to study the polarization of dust particles and the interaction potential of particles with plasma. The results obtained here can be applied in future simulation models as to dust particle motion and their effects on entire plasma.

## Data Availability

The datasets used and/or analysed during the current study available from the corresponding author on reasonable request.

## References

[1] Thomas E, Merlino RL, Rosenberg M (2013). Design criteria for the magnetized dusty plasma experiment. IEEE Trans. Plasma Sci..

[2] Kalita D, Kakati B, Saikia BK, Bandyopadhyay M, Kausik SS (2015). Effect of magnetic field on dust charging and corresponding probe measurement. Phys. Plasmas.

[3] Thomas E, Merlino RL, Rosenberg M (2012). Magnetized dusty plasmas: The next frontier for complex plasma research. Plasma Phys. Control. Fusion.

[4] Tsytovich VN, Sato N, Morfill GE (2003). Note on the charging and spinning of dust particles in complex plasmas in a strong magnetic field. New J. Phys..

[5] Salimullah M, Sandberg I, Shukla PK (2003). Dust charge fluctuations in a magnetized dusty plasma. Phys. Rev. E Stat. Nonlinear Soft Matter Phys..

[6] Mamun AA (1998). Nonlinear propagation of dust-acoustic waves in a magnetized dusty plasma with vortex-like ion distribution. J. Plasma Phys..

[7] Rao NN (1993). Low-frequency waves in magnetized dusty plasmas. J. Plasma Phys..

[8] Kalita D, Kakati B, Kausik SS, Saikia BK, Bandyopadhyay M (2018). Studies on probe measurements in presence of magnetic field in dust containing hydrogen plasma. Eur. Phys. J. D.

[9] Filipič, G. in *University of Ljubljana. Faculty for mathematics and physics*. [Online] http://mafija.fmf.uni-lj.si/seminar/files/2007_2008/Seminar2.pdf.

[10] Buneman O (1959). Dissipation of currents in ionized media. Phys. Rev..

[11] Birdsall CK, Bridges WB (1961). Space-charge instabilities in electron diodes and plasma converters. J. Appl. Phys..

[12] Burger P (1965). Theory of large-amplitude oscillations in the one-dimensional low-pressure cesium thermionic converter. J. Appl. Phys..

[13] Hockney R, Eastwood J (1988). Computer Simulation Using Particles.

[14] Birdsall CK, Fuss D (1997). Clouds-in-clouds, clouds-in-cells physics for many-body plasma simulation. J. Comput. Phys..

[15] Miloch WJ (2015). Simulations of several finite-sized objects in plasma. Procedia Comput. Sci..

[16] Darian D, Miloch WJ, Mortensen M, Miyake Y, Usui H (2019). Numerical simulations of a dust grain in a flowing magnetized plasma. Phys. Plasmas.

[17] Krasheninnikov SI, Smirnov RD, Rudakov DL (2011). Dust in magnetic fusion devices. Plasma Phys. Control. Fusion.

[18] Smirnov RD, Pigarov AY, Rosenberg M, Krasheninnikov SI, Mendis DA (2007). Modelling of dynamics and transport of carbon dust particles in tokamaks. Plasma Phys. Control. Fusion.

[19] Allen JE (1992). Probe theory: The orbital motion approach. Phys. Scr..

[20] Allen JE, Boyd RLF, Reynolds P (1957). The collection of positive ions by a probe immersed in a plasma. Proc. Phys. Soc. Sect. B.

[21] Whipple EC (1981). Potentials of surfaces in space. Rep. Prog. Phys..

[22] Goree J (1994). Charging of particles in a plasma. Plasma Sour. Sci. Technol..

[23] Liu Z, Wang D, Miloshevsky G (2017). Simulation of dust grain charging under tokamak plasma conditions. Nuclear Mater. Energy.

[24] Shukla PK, Mamun AA (2015). Introduction to Dusty Plasma Physics.

[25] Surendra M, Graves DB, Jellum GM (1990). Self-consistent model of a direct-current glow discharge: Treatment of fast electrons. Phys. Rev. A.

[26] Chutov YI, Goedheer WJ (2003). Dusty radio frequency discharges in argon. IEEE Trans. Plasma Sci..

[27] Birdsall CK, Langdon AB (2004). Plasma Physics Via Computer Simulation.

[28] Verboncoeur JP, Langdon AB, Gladd NT (1995). An object-oriented electromagnetic PIC code. Comput. Phys. Commun..

